# A systematic review and meta-analysis of the prevalence of dyslipidaemia among adults in Malaysia

**DOI:** 10.1038/s41598-023-38275-7

**Published:** 2023-07-07

**Authors:** Mohamed-Syarif Mohamed-Yassin, Norhidayah Rosman, Khairatul Nainey Kamaruddin, Hayatul Najaa Miptah, Noorhida Baharudin, Anis Safura Ramli, Suraya Abdul-Razak, Nai Ming Lai, GLOBALDYS Study Investigators

**Affiliations:** 1 Department of Primary Care Medicine, Faculty of Medicine, Universiti Teknologi MARA, Selayang Campus, Jalan Prima Selayang 7, 68100 Batu Caves, Selangor Malaysia; 2 Unit of Pathology, Faculty of Medicine, AIMST University, 08100 Bedong, Kedah Malaysia; 3 Institute of Pathology, Laboratory and Forensic Medicine (I-PPerForM), Universiti Teknologi MARA, Sungai Buloh Campus, Jalan Hospital, 47000 Sungai Buloh, Selangor Malaysia; 4 Cardio Vascular and Lungs Research Institute (CaVaLRI), Hospital Universiti Teknologi MARA (HUiTM), Jalan Hospital, 47000 Sungai Buloh, Selangor Malaysia; 5 School of Medicine, Taylor’s University, 1, Jalan Taylor’s, 47500 Subang Jaya, Selangor Malaysia

**Keywords:** Dyslipidaemias, Dyslipidaemias, Epidemiology

## Abstract

Dyslipidaemia is an established cardiovascular risk factor. This study aimed to determine the pooled prevalence of dyslipidaemia in Malaysian adults. A systematic review and meta-analysis of all cross-sectional, longitudinal observational studies which reported the prevalence of elevated total cholesterol (TC), low-density lipoprotein cholesterol (LDL-c), triglycerides (TG), and reduced high-density lipoprotein cholesterol (HDL-c) in adults 18 years old and older, was conducted. A comprehensive search of PubMed and Cochrane Central Register of Controlled Trials (which included Medline, EMBASE and major trial registers) from inception to October 18, 2022, was performed. Risk-of-bias was evaluated using the Johanna-Briggs Institute Prevalence Critical Appraisal Tool, while certainty of evidence was assessed using an adapted version of the Grading of Recommendations Assessment, Development, and Evaluation (GRADE) framework. Random-effects meta-analyses were performed using MetaXL. This report follows the PRISMA reporting guidelines. The protocol was registered with PROSPERO (CRD42020200281). 26 556 studies were retrieved and 7 941 were shortlisted initially. From this, 70 Malaysian studies plus two studies from citation searching were shortlisted; 46 were excluded, and 26 were included in the review (n = 50 001). The pooled prevalence of elevated TC (≥ 5.2 mmol/L), elevated LDL-c (≥ 2.6 mmol/L), elevated TG (≥ 1.7 mmol/L), and low HDL-c (< 1.0 mmol/L in men and < 1.3 mmol/L in women) were 53% (95% CI 39–67%, I^2^ = 100%), 73% (95% CI 50–92%, I^2^ = 100%), 36% (95% CI 32–40%, I^2^ = 96%), and 40% (95% CI 25–55%, I^2^ = 99%), respectively. This review found that the prevalence of all dyslipidaemia subtypes is high in Malaysian adults. Ongoing efforts to reduce cardiovascular diseases in Malaysia should integrate effective detection and treatment of dyslipidaemia.

## Introduction

Dyslipidaemia is defined as lipid disorders with either one or any combination of elevated total cholesterol (TC), elevated low-density lipoprotein cholesterol (LDL-c), elevated triglycerides (TG) or low high-density lipoprotein cholesterol (HDL-c)^1^. It is an established cardiovascular risk factor. Large-scale randomised trials concluded that coronary mortality and all-cause mortality reduction can be achieved by effective treatment of dyslipidaemia^2^. The 5-year incidence of major coronary events, stroke, and coronary revascularisation can be decreased to about one fifth with a 1 mmol/L LDL-c reduction through statin therapy^2,3^.

Prevalence is defined as the proportion of a group of people that is affected by a clinical condition^4^. Prevalence estimates are used to estimate the burden of diseases, thus guiding the prioritisation of interventions, development of clinical practice guidelines, and research^5^. As these estimates depict changes and trends in the outcome of interest over a period of time, they are helpful to assess the outcome of health interventions^5^.

The World Health Organization (WHO) estimated in 2008 that the hypercholesterolaemia (elevated TC) prevalence in adults were 23.1% in Africa, 30.3% in South East Asia, 47.7% in America, and 53.7% in Europe^6^. However, there is no published systematic review on the worldwide dyslipidaemia prevalence in adults. Hence, we had undertaken a large-scale systematic review and meta-analysis entitled the GLOBAL prevalence of DYSlipidaemia in adults (GLOBALDYS) study, to determine the global prevalence of dyslipidaemia in adults 18 years old and older.

For Malaysia, a country in the Western Pacific region, ischaemic heart disease had consistently been the principal cause of death in the past decade^7–11^. Many studies had reported on the prevalence of the major atherosclerotic cardiovascular disease (ASCVD) risk factors with a recent meta-analysis reporting that the pooled prevalence of diabetes mellitus in Malaysia was 14.39%^12^. However, to date, there is no published data on the pooled prevalence of dyslipidaemia in Malaysian adults. To address this gap, we performed a systematic review and meta-analysis of the prevalence of dyslipidaemia in Malaysian adult populations to guide prevention, detection, and control strategies, as the first subproject of the GLOBALDYS study.

## Methods

### Systematic review protocol and registration

This report was prepared in accordance with the Preferred Reporting Items for Systematic Reviews and Meta-Analyses (PRISMA) statement, (eTable 1)^13^. The protocol for the systematic review was registered with PROSPERO (number CRD42020200281)^14^ and published elsewhere^15^.

### Search strategy and selection criteria

We searched PubMed/MEDLINE, Cochrane Central Register of Controlled Trials (CENTRAL), which covered Medline, EMBASE and major trial registries including the WHO International Trial Registry Platform and ClinicalTrials.gov for ongoing studies. The search strategy combined the search term “prevalence” and several terms for dyslipidaemia which included “dyslipidemia”, “hyperlipidemia”, “hypercholesterolaemia”, “hypertriglyceridemia”, and “lipid disorder”. This main search strategy was utilized in PubMed and adapted to the other databases (eTable 2). We also manually searched the reference list of eligible articles to find other relevant articles.

The searches were performed from inception to October 18, 2022. Given the large amount of search results, the repeated search for this manuscript was performed by reviewing search results from PubMed/MEDLINE’s saved search alert with a more focused search strategy (eTable 3). There were no restrictions applied to language and publication period.

We included all cross-sectional, longitudinal observational studies which reported the prevalence of elevated serum TC, elevated LDL-c, elevated TG, or low HDL-c in adults 18 years old and above. We excluded publications on children and familial hypercholesterolaemia. Editorials, commentaries, reviews, letters, case series with less than 50 patients, and studies without primary data or explicit description of methods were also excluded. For duplicate publications of the same studies, we selected the reports with the largest sample size.

### Study selection, data abstraction, and quality appraisal

Title, abstract, and full-text screening were performed by two independent reviewers in two teams (M.S.M.Y. and N.R.; K.N.K., and H.N.M.). ASReview, an open-source machine learning-aided pipeline with active learning software was utilized during the first round of study selection^16^. Disagreements were resolved through discussion leading to a consensus.

A pro forma specifically designed for this review was used to extract the following data: first author’s name, publication year, study design, country, locality (rural vs. urban), setting (community or hospital-based), sample size, mean/median age, age range, proportion of men/women, any disease specific to the study population, dyslipidaemia subtypes included (i.e. elevated TC, elevated LDL-c, elevated TG, low HDL-c), diagnostic cut-off levels and the number of participants with dyslipidaemia. The cut-offs for the diagnosis of each category of dyslipidaemia were chosen mainly based on the Malaysian Clinical Practice Guideline for Management of Dyslipidaemia 2017^1^: elevated TC (≥ 5.2 mmol/L), elevated LDL-c (≥ 2.6 mmol/L), elevated TG (≥ 1.7 mmol/L), and low HDL-c (< 1.0 mmol/L in men and < 1.3 mmol/L in women).

Risk of bias were assessed using the Joanna Briggs Institute Prevalence Critical Appraisal Tool^17^. This tool consists of nine questions with four standard answer options (yes/no/unclear/not applicable). The overall appraisal consists of three answer options (include/exclude/seek further info) based on the rater’s judgement. Risk of bias assessment was performed by one reviewer (M.S.M.Y.). Then, another reviewer (N.R.) randomly selected and independently performed risk of bias assessment on three (10%) out of the 26 studies.

We used an adapted version of the GRADE tool^18^ to assess certainty of evidence as high, moderate, low, or very low^19^. The components of this tool include study limitations, imprecision, indirectness, inconsistency, and publication bias.

### Statistical analysis

We first performed a narrative synthesis of the study results, and performed meta-analysis to obtain elevated TC (≥ 5.2 mmol/L), elevated LDL-c (≥ 2.6 mmol/L), elevated TG (≥ 1.7 mmol/L), and low HDL-c (< 1.0 mmol/L in men and < 1.3 mmol/L in women) synthesised point estimate of prevalence with its 95% confidence intervals (CI) using the MetaXL software version 5.3 (EpiGear International, Queensland, Australia). We transformed all prevalence estimates using the Freeman-Tukey transformation (arcsine square root transformation) to minimize the influence from studies with extreme prevalence estimates on the overall estimate^20^. The point estimate and 95% CI was then back-transformed and pooled using random effects model meta-analysis^21^.

The degree of heterogeneity in the estimates among studies was measured using the I^2^ statistics, with an adopted cut-off of 75% indicating a substantial degree of heterogeneity^22^. The possible contributors of heterogeneity including population characteristics and study settings were assessed, and studies were divided into appropriate subgroups for pooling of results.

We performed sensitivity analyses by excluding studies judged as having a high risk of bias.

The traditional funnel plot had been found to result in very limited sensitivity for publication bias assessment when used for meta-analysis with less than ten studies^23^. It also resulted in non-interpretability when used assessing publication bias in meta-analysis of prevalence studies^24^. Hence, publication bias was assessed using the Doi plot and LFK index^25^, which had been reported as better methods to detect and quantify asymmetry. The Doi plot is inspected visually similar to the classic funnel plot. For the LFK index, values beyond ± 1 are deemed consistent with asymmetry^25^.

We assessed inter-rater agreement for study inclusion, data extraction, and risk of bias analysis using Cohen’s kappa coefficient (κ)^26^.

## Results

### Study selection

**Figure 1:**
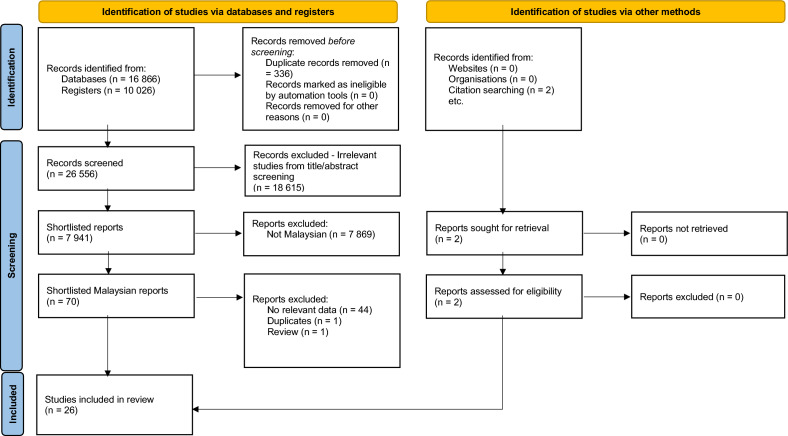
Study flow diagram. From: Page MJ, McKenzie JE, Bossuyt PM, Boutron I, Hoffmann TC, Mulrow CD, et al. The PRISMA 2020 statement: an updated guideline for reporting systematic reviews. BMJ 2021; 372:n71. https://doi.org/10.1136/bmj.n71. For more information, visit: http://www.prisma-statement.org/.

The initial search performed on PubMed found 16 866 studies. Next, the CENTRAL database search revealed 10 026 studies. The total of 26 892 studies were imported into EndNote^27^ and deduplicated, leaving 26 556 studies. These 26 556 studies were then imported into ASReview. M.S.M.Y. reviewed 16 425 out of the 26 556 studies and categorized 8 883 as potentially relevant, 7 542 as irrelevant, with 10 131 unreviewed. Next, K.N.K. and H.N.M. reviewed the 7 542 irrelevant and 10 131 unreviewed studies. From this second round of review, a total of 138 studies were categorized as potentially relevant, making the total of potentially relevant studies 9 021. M.S.M.Y. and N.R. then performed a third round of review of the 9 021 studies classified as potentially relevant. From this final round of review, 7 941 studies were categorized as potentially relevant and 1 080 were categorized as irrelevant (Fig. 1).

The 7 941 potentially relevant studies were conducted across the world. The review team divided the project into a series of studies focusing on the WHO regions (Western Pacific, South-East Asia, Europe, Americas, Eastern Mediterranean, and Africa) and individual countries of the world. The current review is the first country-level review of the series, focusing on the prevalence of dyslipidaemia in Malaysia.

70 potentially relevant Malaysian studies and two other studies found via citation searching, were assessed by two review authors (M.S.M.Y. and N.R.) independently. From this, 27 studies were selected for inclusion in the review. Both reviewers had perfect agreement on the selection of studies (κ = 1.00). Data was then extracted by M.S.M.Y. For one study, the total participants with elevated TC could not be derived^28^. The lead author of the study was contacted via email but unfortunately, he was unable to provide the required data as he no longer had access to it. Hence, this study was excluded from our systematic review. Next, N.R. independently crosschecked the data extracted from the 26 eligible studies and discrepancies were resolved through discussion with M.S.M.Y. The final inter-rater agreement for data extraction was high (level of agreement = 100%; κ = 1.00).

These 26 studies involving 50 001 participants (eTable 4) were included for meta-analysis of prevalence^29–54^. From these, 14 studies reported on elevated TC^29–31,33,35,37,39–41,45,46,48,50,53^, five studies on elevated LDL-c^33,37,44,46,53^, 16 studies on elevated TG^30,32–34,36,38,42,43,47–54^, and six studies on low HDL-c^34,36,47,49,52,54^. Overall, the studies were published between 1996 and 2021. There were slightly more community-based studies (15 studies)^29–32,35,40,41,45,47–52,54^ compared to clinic or hospital-based ones (11 studies)^33,34,36–39,42–44,46,53^.

M.S.M.Y. classified six studies (23.1%)^30,32,42,43,53,54^ as having high risk of bias and 20 studies (76.9%)^29,31,33–41,44–52^ as having low risk of bias (eTable 4). N.R. randomly selected three studies (~ 10% from the total of 26 studies) and independently assessed their risk of bias. The inter-rater agreement between M.S.M.Y. and N.R. for this step was high (level of agreement = 100%; κ = 1.00).

### Prevalence of dyslipidaemia subtypes

**Figure 2:**
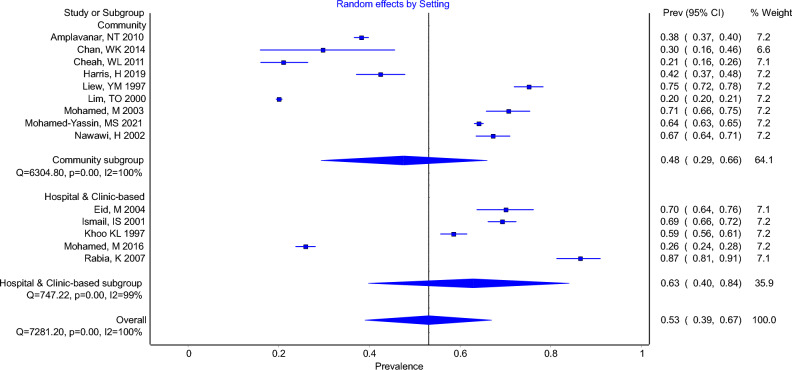
Forest Plot Showing Prevalence of Elevated Total Cholesterol (TC ≥ 5.2 AND > 5.2) in Community-based Studies and Hospital or Clinic-based Studies.

The overall prevalence of elevated TC with a cut-off of at least 5.2 mmol/L was 53% (95% CI 39–67%, I^2^ = 100%). The pooled prevalence for elevated TC in community-based studies was 48% (95% CI 29–66%, I^2^ = 100%) while the pooled prevalence for elevated TC in hospital or clinic-based patients was 63% (95% CI 40–84%, I^2^ = 99%) (Fig. 2).

**Figure 3:**
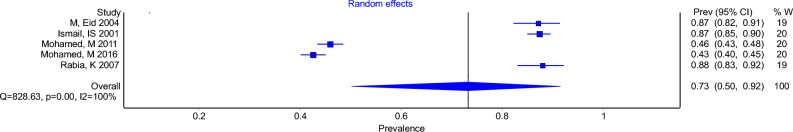
Forest Plot Showing Prevalence of Elevated LDL-cholesterol (LDL-c ≥ 2.6) in Hospital or Clinic-based Studies.

With a cut-off of at least 2.6 mmol/L, the overall prevalence of elevated LDL-c was 73% (95% CI 50–92%, I^2^ = 100%), which consisted of only hospital or clinic-based studies (Fig. 3).

**Figure 4:**
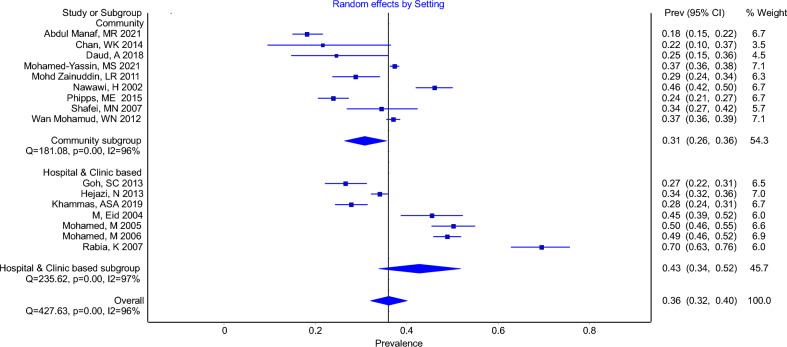
Forest Plot Showing Prevalence of Elevated Triglycerides (TG ≥ 1.7 & > 1.7) in Community-based Studies and Hospital or Clinic-based Studies.

The overall pooled prevalence of elevated TG was 36% (95% CI 32–40%, I^2^ = 96%), using a cut-off of at least 1.7 mmol/L. The pooled prevalence for elevated TG in community-based studies and hospital or clinic-based studies were 31% (95% CI 26–36%, I^2^ = 96%) and 43% (95% CI 34–52%, I^2^ = 97%), respectively (Fig. 4).

**Figure 5:**
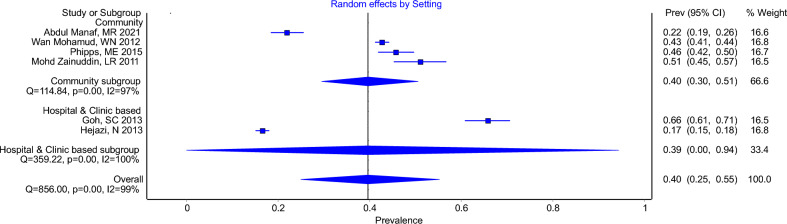
Forest Plot Showing Prevalence of Low HDL-cholesterol (HDL-c < 1 in men & < 1.3 women) in Community-based Studies and Hospital or Clinic-based Studies.

Using a cut-off of less than 1 mmol/L and 1.3 mmol/L in women and men, respectively, the overall pooled prevalence of low HDL-c was 40% (95% CI 25–55%, I^2^ = 99%). For low HDL-c in community-based studies and hospital or clinic-based studies, the pooled prevalence were 40% (95% CI 30–51%, I^2^ = 97%) and 39% (95% CI 0–94%, I^2^ = 100%), respectively (Fig. 5).

Sensitivity analyses for each dyslipidaemia subtype were performed. For elevated TC, we excluded two studies judged as having high risk of bias^30,53^. The overall pooled prevalence for elevated TC decreased slightly to 52% (95% CI 37%–67%, I^2^ = 100%). The pooled prevalence for elevated TC in community-based studies increased slightly to 50% (95% CI 30–69%, I^2^ = 100%) while the pooled prevalence for elevated TC in hospital or clinic-based patients decreased to 56% (95% CI 32–80%, I^2^ = 100%) (eFig. 1).

For elevated LDL-c, when a study with high risk of bias^53^ was removed, the overall pooled prevalence decreased to 69% (95% CI 43–90%, I^2^ = 100%) (eFig. 2).

For elevated TG, we removed six studies judged as having high risk of bias^30,32,42,43,53,54^. Following this, the overall pooled prevalence of elevated TG decreased slightly to 33% (95% CI 29–37%, I^2^ = 96%). The pooled prevalence in community-based studies increased slightly to 33% (95% CI 27–38%, I^2^ = 97%), while the prevalence for hospital or clinic-based studies dropped to 33% (95% CI 27–39%, I^2^ = 90%) (eFig. 3).

For low HDL-c, when a study with high risk of bias^54^ was excluded, the pooled prevalence for community-based studies decreased to 36% (95% CI 24–49%, I^2^ = 98%). The overall pooled prevalence also decreased to 37% (95% CI 21–55%, I^2^ = 100%) (eFig. 4). A summary table of the prevalence of each dyslipidaemia subtype pre and post sensitivity analyses is presented in eTable 5.

### Publication bias assessment

The Doi plots and LFK indices for elevated TC (LFK index = 5.65) and elevated LDL-c (LFK index = 5.35) indicated major asymmetry in favour of studies reporting higher prevalence of these dyslipidaemia subtypes (eFigs. 5 and 6). In contrast, the Doi plots and LFK indices for elevated TG and low HDL-c were consistent with no asymmetry (LFK index = − 0.83), and minor asymmetry (LFK index = 1.12), respectively (eFigs. 7 and 8).

### Certainty of evidence: GRADE

We judged the overall quality of the available evidence on the pooled prevalence of the four dyslipidaemia subtypes as of low certainty (eTable 6). This judgement was made because we included six studies with high risk of bias and there was substantial heterogeneity.

## Discussion

As far as we are aware, this is the first report of pooled dyslipidaemia prevalence in Malaysian adults. From this review which included 26 community-based and hospital or clinic-based studies involving 50 001 participants, high prevalence of all dyslipidaemia subtypes were observed (elevated TC = 53%, elevated LDL-c = 73%, elevated TG = 36%, reduced HDL-c = 40%).

As expected, the pooled prevalence of elevated TC and elevated TG were higher in the hospital or clinic-based studies. However, we found that the pooled prevalence of reduced HDL-c was almost similar between community-based and hospital or clinic-based studies. A meta-analysis of dyslipidaemia prevalence in Africa reported similar findings for elevated TC and reduced HDL-c, but not for elevated TG^55^.

The prevalence of elevated TC was higher compared to meta-analyses findings from Iran (42%) and Nigeria (38%), but lower compared to Portugal (56.7%)^56–58^. The prevalence of elevated LDL-c was also higher while reduced HDL-c was lower compared to a review from Iran (elevated LDL-c = 40%, reduced HDL-c = 43%)^56^. The very high prevalence of elevated LDL-c (73%) should be a cause for concern as numerous genetic, epidemiologic, and clinical studies had consistently found that elevated LDL-c is a cause of atherosclerotic cardiovascular disease^59–68^.

The strengths of this study include the systematic and comprehensive search using reproducible and rigorous methodological procedures, based on a preregistered and published protocol. Another strength is the utilization of robust statistical methods to pool eligible studies and the subsequent systematic synthesis of the pooled data.

The results of this study, however, need to be interpreted in the context of some limitations. Firstly, consistent with the progression of clinical practice guidelines, a variety of cut-offs were used to define dyslipidaemia in published studies. For this meta-analysis, the cut-offs chosen for elevated TC and elevated TG were consistent with the Malaysian Clinical Practice Guidelines on Management of Dyslipidaemia 2017^1^. In clinical practice, the cut-off for elevated LDL-c depends on an individual’s overall cardiovascular risk: > 3.4 mmol/L, > 2.6 mmol/L, and > 1.8 mmol/L for moderate, high, and very high cardiovascular risk, respectively. As we did not have the data on our participants’ cardiovascular risk score or the means to derive it, we decided to choose > 2.6 mmol/L, taking into consideration that we included participants from community-based and hospital or clinic-based studies. For low HDL-c, a variety of cut-offs were used in the studies that we found as there were different cut-offs for men and women. We decided to choose < 1.0 mmol/L in men and < 1.3 mmol/L in women as these were the most frequently used ones.

Secondly, there was substantial heterogeneity found between studies with all I^2^ levels of more than 90%. This was not explained by subgroup analysis based on study settings (community-based studies or hospital or clinic-based studies). Meta-regression was not performed as no suitable covariates besides the study setting were identified. A literature review showed that this level of heterogeneity is consistent with many other meta-analyses of prevalence studies^12,56,57,69–73^. This finding was commented upon by Imrey who said that “meta-analyses addressing the incidence or prevalence of a phenomenon in diverse environments may assemble highly heterogenous studies”^74^.

Thirdly, six studies deemed to have high risk of bias were included in this meta-analysis. To address these, sensitivity analyses which excluded these studies were performed. Although these analyses found some changes to the pooled prevalence of the different dyslipidaemia subtypes, these changes were not substantial. Also, the level of heterogeneity remained high even with the exclusion of these studies. Our next limitation is that we did not report on other forms of dyslipidaemia such as elevated non-HDL cholesterol (non-HDL-c), as there was only one study which reported on the prevalence of this dyslipidaemia subtype^48^. Finally, there is significant publication bias in favour of studies reporting higher prevalence of elevated TC and elevated LDL-c.

Findings from our study suggest that urgent and continuous public health measures are needed to address the high prevalence of all dyslipidaemia subtypes among Malaysian adults. Health campaigns to educate the public on dyslipidaemia and healthy eating habits such as reducing trans-fat intake should be intensified. A recent local study concluded that there were still gaps in knowledge and practice in dyslipidaemia management even among doctors pursuing postgraduate primary care qualifications^75^. Hence, all healthcare professionals should be regularly updated on the recommendations for prevention and treatment of dyslipidaemia based on the latest Malaysian Clinical Practice Guidelines on Management of Dyslipidaemia^1^ via continuing professional education activities to reduce clinical inertia. This is because one of the reasons for clinical inertia was found to be a lack of education, training and practice organization aimed at achieving therapeutic goals^76^. Another factor which may affect dyslipidaemia management is the heavy workload of doctors especially in the public sector, which handles the majority of the Malaysian national healthcare workload^77^. It is hoped that the latest initiatives by the Malaysian government which is the development of the Health White Paper and the formation of a Health Reform Commission will help to address this chronic maldistribution of human resource and workload between the public and private sectors^78^. The increased availability of generic versions of moderate and high-potency statins such as atorvastatin and rosuvastatin should hopefully decrease the barrier to prescribing and optimizing statin therapy for those indicated^79^. Along with appropriate dietary choices and exercise, these are effective medications to lower LDL-c and TG, as well as increasing HDL-c^3,80–83^.

Researchers in the field should use the dyslipidaemia cut-off levels recommended by the Malaysian Clinical Practice Guidelines on Management of Dyslipidaemia^1^, to ensure a more uniformed definition of dyslipidaemia. Future research should also incorporate the measurement of non-HDL-c levels. It is simply calculated by deducting HDL-c from TC. At no extra cost, it measures all atherogenic apolipoprotein B-containing lipoproteins including LDL-c^84^. The current literature supports the measurement of non-HDL-c levels as it has been found to be a better predictor of coronary heart disease risk than LDL-c alone^85,86^.

## Conclusions

This study found that the prevalence of all dyslipidaemia subtypes is high in the adult populations in Malaysia. Urgent public health measures are needed to address this established cardiovascular risk factor. These efforts include improving access to laboratory testing, educating physicians in dyslipidaemia care, and facilitating access to lipid-modifying therapies. Appropriate dietary choices and exercise, along with lipid-lowering medications should be prescribed for those with dyslipidaemia, if indicated.

## Data sharing

Data collected for this review including search results and study protocol, will be made available to others, from the publication date, by emailing the corresponding author.

### Supplementary Information

Supplementary Information.

## Data Availability

Data collected for this review including search results and study protocol, will be made available to others, from the publication date, by emailing the corresponding author.
