# Author Correction: A systematic review and meta-analysis of the prevalence of dyslipidaemia among adults in Malaysia

**DOI:** 10.1038/s41598-023-41219-w

**Published:** 2023-09-07

**Authors:** Mohamed-Syarif Mohamed-Yassin, Norhidayah Rosman, Khairatul Nainey Kamaruddin, Hayatul Najaa Miptah, Noorhida Baharudin, Anis Safura Ramli, Suraya Abdul-Razak, Nai Ming Lai, GLOBALDYS Study Investigators

**Affiliations:** 1 Department of Primary Care Medicine, Faculty of Medicine, Universiti Teknologi MARA, Selayang Campus, Jalan Prima Selayang 7, 68100 Batu Caves, Selangor Malaysia; 2 Unit of Pathology, Faculty of Medicine, AIMST University, 08100 Bedong, Kedah Malaysia; 3 Institute of Pathology, Laboratory and Forensic Medicine (I-PPerForM), Universiti Teknologi MARA, Sungai Buloh Campus, Jalan Hospital, 47000 Sungai Buloh, Selangor Malaysia; 4 Cardio Vascular and Lungs Research Institute (CaVaLRI), Hospital Universiti Teknologi MARA (HUiTM), Jalan Hospital, 47000 Sungai Buloh, Selangor Malaysia; 5 School of Medicine, Taylor’s University, 1, Jalan Taylor’s, 47500 Subang Jaya, Selangor Malaysia

Correction to: *Scientific Reports* 10.1038/s41598-023-38275-7, published online 07 July 2023

The original version of this Article contained errors caused by errors in data extraction. This has now been corrected.

As a result, in the Abstract section,

“…and low HDL-c (< 1.0 mmol/L in men and < 1.3 mmol/L in women) were 52% (95% CI 32–71%, I^2^ = 100%)…”

now reads:

“…and low HDL-c (< 1.0 mmol/L in men and < 1.3 mmol/L in women) were 53% (95% CI 39–67%, I^2^ = 100%)…”

Also, in the Results section, under the subheading ‘Prevalence of dyslipidaemia subtypes’,

“The overall prevalence of elevated TC with a cut-off of at least 5.2 mmol/L was 52% (95% CI 32%–71%, I^2^ = 100%). The pooled prevalence for elevated TC in community-based studies was 45% (95% CI 19–72%, I^2^ = 100%)... The overall pooled prevalence for elevated TC decreased slightly to 50% (95% CI 29%–71%, I^2^ = 100%). The pooled prevalence for elevated TC in community-based studies increased slightly to 47% (95% CI 19–75%, I^2^ = 100%)…”

now reads:

“The overall prevalence of elevated TC with a cut-off of at least 5.2 mmol/L was 53% (95% CI 39%–67%, I^2^ = 100%). The pooled prevalence for elevated TC in community-based studies was 48% (95% CI 29–66%, I^2^ = 100%)... The overall pooled prevalence for elevated TC decreased slightly to 52% (95% CI 37%–67%, I^2^ = 100%). The pooled prevalence for elevated TC in community-based studies increased slightly to 50% (95% CI 30–69%, I^2^ = 100%)…”

And in the same section, under the subheading ‘Publication bias assessment’,

‘elevated TC (LFK index = 4.77)’

now reads:

‘elevated TC (LFK index = 5.65)’.

Further, in the Discussion section,

‘elevated TC = 52%’

now reads:

‘elevated TC = 53%’.

Moreover, in Figure 2, several values under ‘Prev (95% CI)’, ‘% Weight’, and ‘Q’ were incorrect. The original Figure 2 and the accompanying legend appear here.

**Figure 2:**
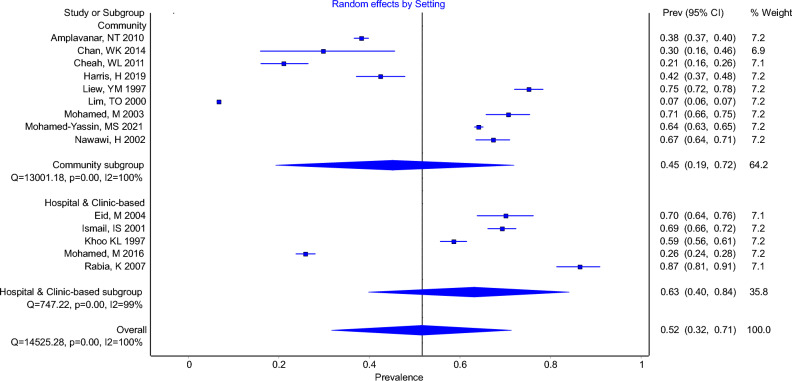
Forest Plot Showing Prevalence of Elevated Total Cholesterol (TC ≥ 5.2 AND > 5.2) in Community-based Studies and Hospital or Clinic-based Studies.

Finally, the Supplementary Information file contained errors in eTable 4, eTable 5, eFigure 1, and eFigure 5. The original Supplementary Information file is provided below.

The original Article and its accompanying Supplementary Information have been corrected.

### Supplementary Information

Supplementary Information.

