# Supplementary material for: A systematic review and meta-analysis of the prevalence of dyslipidaemia among adults in Malaysia
Source: Sci Rep. 2023 Jul 7;13:11036. doi: 10.1038/s41598-023-38275-7 (PMC10328969; doi:10.1038/s41598-023-38275-7)
Supplement: Supplementary file 1 — Supplementary Information. [file 41598_2023_38275_MOESM1_ESM.pdf]

Supplemental Online Content

Prevalence of Dyslipidaemia Among Adults in Malaysia - A Systematic Review and Meta-Analysis

Mohamed-Syarif Mohamed-Yassin, Norhidayah Rosman, Khairatul Nainey Kamaruddin, Hayatul Najaa Miptah, Noorhida Baharudin, Anis Safura Ramli, Suraya Abdul-Razak, Nai Ming Lai on behalf of the GLOBALDYS Study Investigators

eTable 1. Preferred Reporting Items for Systematic Reviews and Meta-Analyses (PRISMA) checklist

eTable 2. Search Strategy

eTable 3. Adapted Search Strategy

eTable 4. Characteristics of included studies

eTable 5. Sensitivity analyses findings summary

eTable 6. GRADE assessment for the studies included in the synthesis with meta-analysis

eFigure 1. Sensitivity analysis – Pooled prevalence of elevated TC excluding studies with high risk of bias

eFigure 2. Sensitivity analysis – Pooled prevalence of elevated LDL-c excluding study with high risk of bias

eFigure 3. Sensitivity analysis – Pooled prevalence of elevated TG excluding studies with high risk of bias

eFigure 4. Sensitivity analysis – Pooled prevalence of low HDL-c excluding study with high risk of bias

eFigure 5. Doi plot and LFK index for elevated TC

eFigure 6. Doi plot and LFK index for elevated LDL-c

eFigure 7. Doi plot and LFK index for elevated TG

eFigure 8. Doi plot and LFK index for low HDL-c

eReferences

This supplemental material has been provided by the authors to give readers additional information about their work.

PRISMA 2020 Checklist

Section and Topic	Item #	Checklist item	Location where item is reported
**TITLE**			
Title	1	Identify the report as a systematic review.	1
**ABSTRACT**			
Abstract	2	See the PRISMA 2020 for Abstracts checklist.	1
**INTRODUCTION**			
Rationale	3	Describe the rationale for the review in the context of existing knowledge.	1,2,3
Objectives	4	Provide an explicit statement of the objective(s) or question(s) the review addresses.	3
**METHODS**			
Eligibility criteria	5	Specify the inclusion and exclusion criteria for the review and how studies were grouped for the syntheses.	4,5
Information sources	6	Specify all databases, registers, websites, organisations, reference lists and other sources searched or consulted to identify studies. Specify the date when each source was last searched or consulted.	4
Search strategy	7	Present the full search strategies for all databases, registers and websites, including any filters and limits used.	eTable 2 and eTable 3
Selection process	8	Specify the methods used to decide whether a study met the inclusion criteria of the review, including how many reviewers screened each record and each report retrieved, whether they worked independently, and if applicable, details of automation tools used in the process.	5
Data collection process	9	Specify the methods used to collect data from reports, including how many reviewers collected data from each report, whether they worked independently, any processes for obtaining or confirming data from study investigators, and if applicable, details of automation tools used in the process.	5,7,8
Data items	10a	List and define all outcomes for which data were sought. Specify whether all results that were compatible with each outcome domain in each study were sought (e.g. for all measures, time points, analyses), and if not, the methods used to decide which results to collect.	5
	10b	List and define all other variables for which data were sought (e.g. participant and intervention characteristics, funding sources). Describe any assumptions made about any missing or unclear information.	5
Study risk of bias assessment	11	Specify the methods used to assess risk of bias in the included studies, including details of the tool(s) used, how many reviewers assessed each study and whether they worked independently, and if applicable, details of automation tools used in the process.	5,6
Effect measures	12	Specify for each outcome the effect measure(s) (e.g. risk ratio, mean difference) used in the synthesis or presentation of results.	6
Synthesis methods	13a	Describe the processes used to decide which studies were eligible for each synthesis (e.g. tabulating the study intervention characteristics and comparing against the planned groups for each synthesis (item #5)).	5,6
	13b	Describe any methods required to prepare the data for presentation or synthesis, such as handling of missing summary statistics, or data conversions.	6
	13c	Describe any methods used to tabulate or visually display results of individual studies and syntheses.	6
	13d	Describe any methods used to synthesize results and provide a rationale for the choice(s). If meta-analysis was performed, describe the model(s), method(s) to identify the presence and extent of statistical heterogeneity, and software package(s) used.	6
	13e	Describe any methods used to explore possible causes of heterogeneity among study results (e.g. subgroup analysis, meta-regression).	6
	13f	Describe any sensitivity analyses conducted to assess robustness of the synthesized results.	7
Reporting bias assessment	14	Describe any methods used to assess risk of bias due to missing results in a synthesis (arising from reporting biases).	7

PRISMA 2020 Checklist

Section and Topic	Item #	Checklist item	Location where item is reported
Certainty assessment	15	Describe any methods used to assess certainty (or confidence) in the body of evidence for an outcome.	6
**RESULTS**			
Study selection	16a	Describe the results of the search and selection process, from the number of records identified in the search to the number of studies included in the review, ideally using a flow diagram.	7-9
	16b	Cite studies that might appear to meet the inclusion criteria, but which were excluded, and explain why they were excluded.	8
Study characteristics	17	Cite each included study and present its characteristics.	9 & eTable 4
Risk of bias in studies	18	Present assessments of risk of bias for each included study.	9 & eTable 4
Results of individual studies	19	For all outcomes, present, for each study: (a) summary statistics for each group (where appropriate) and (b) an effect estimate and its precision (e.g. confidence/credible interval), ideally using structured tables or plots.	9,10 & eTable 4
Results of syntheses	20a	For each synthesis, briefly summarise the characteristics and risk of bias among contributing studies.	9,10
	20b	Present results of all statistical syntheses conducted. If meta-analysis was done, present for each the summary estimate and its precision (e.g. confidence/credible interval) and measures of statistical heterogeneity. If comparing groups, describe the direction of the effect.	9,10, Figures 2-5
	20c	Present results of all investigations of possible causes of heterogeneity among study results.	10
	20d	Present results of all sensitivity analyses conducted to assess the robustness of the synthesized results.	10,11, eTable 5, eFigures 1-4
Reporting biases	21	Present assessments of risk of bias due to missing results (arising from reporting biases) for each synthesis assessed.	NA
Certainty of evidence	22	Present assessments of certainty (or confidence) in the body of evidence for each outcome assessed.	11 & eTable 6
**DISCUSSION**			
Discussion	23a	Provide a general interpretation of the results in the context of other evidence.	11,12
	23b	Discuss any limitations of the evidence included in the review.	12,13
	23c	Discuss any limitations of the review processes used.	13,14
	23d	Discuss implications of the results for practice, policy, and future research.	14,15
**OTHER INFORMATION**			
Registration and protocol	24a	Provide registration information for the review, including register name and registration number, or state that the review was not registered.	4
	24b	Indicate where the review protocol can be accessed, or state that a protocol was not prepared.	4
	24c	Describe and explain any amendments to information provided at registration or in the protocol.	NA
Support	25	Describe sources of financial or non-financial support for the review, and the role of the funders or sponsors in the review.	7
Competing interests	26	Declare any competing interests of review authors.	16
Availability of	27	Report which of the following are publicly available and where they can be found: template data collection forms; data extracted from included	16

Section and Topic	Item #	Checklist item	Location where item is reported
data, code and other materials		studies; data used for all analyses; analytic code; any other materials used in the review.	

eTable 1. Preferred Reporting Items for Systematic Reviews and Meta-Analyses (PRISMA) checklist

From: Page MJ, McKenzie JE, Bossuyt PM, Boutron I, Hoffmann TC, Mulrow CD, et al. The PRISMA 2020 statement: an updated guideline for reporting systematic reviews. BMJ 2021;372:n71. doi: 10.1136/bmj.n71

For more information, visit: http://www.prisma-statement.org/

Search strategy PubMed/MEDLINE (adapted for CENTRAL)	
Search	Search terms
1	dyslipid*[Title/Abstract]
2	dyslipidemia[MeSH Terms]
3	hyperlipid*[Title/Abstract]
4	hyperlipidemia[MeSH Terms]
5	hypercholesterol*[Title/Abstract]
6	essential hypercholesterolemia[MeSH Terms]
7	hypertriglycerid*[Title/Abstract]
8	hypertriglyceridemia[MeSH Terms]
9	"lipid disorder"[Title/Abstract]
10	#1 OR #2 OR #3 OR #4 OR #5 OR #6 OR #7 OR #8 OR #9
11	prevalence[Title/Abstract]
12	prevalence[MeSH Terms]
13	#11 OR #12
14	#10 AND #13

eTable 2. Search Strategy

Search strategy PubMed/MEDLINE (adapted for CENTRAL)	
Search	Search terms
1	dyslipid*[Title/Abstract]
2	dyslipidemia[MeSH Terms]
3	hyperlipid*[Title/Abstract]
4	hyperlipidemia[MeSH Terms]
5	hypercholesterol*[Title/Abstract]
6	essential hypercholesterolemia[MeSH Terms]
7	hypertriglycerid*[Title/Abstract]
8	hypertriglyceridemia[MeSH Terms]
9	“lipid disorder”[Title/Abstract]
10	#1 OR #2 OR #3 OR #4 OR #5 OR #6 OR #7 OR #8 OR #9
11	prevalence[Title/Abstract]
12	prevalence[MeSH Terms]
13	#11 OR #12
14	malaysia[MeSH Terms]
15	malaysia[All Fields]
16	#14 OR #15
17	#10 AND #13 AND #16

eTable 3. Adapted Search Strategy Table

First author name	Publication year	N	Cases	Prevalence	Locality (Rural vs Urban)	Setting (Community vs Hospital/Clinic-based	Mean/Median age & range	Proportion Men/Women	Specific Disease/ Population	Dyslipidaemia subtypes	Diagnostic cutoff level	Quality-Risk of bias
Amplavanar, NT[1]	2010	3772	1442 -TC	40.2% -TC	Urban	Community	46.9 (SD 7.86)	64.7%/ 35.3%	No	TC	TC ≥5.2	Low
Abdul Manaf, MR[2]	2021	538	98, 118 - TG, HDL-c	18.2%, 21.9% -TG, HDL-c	Urban	Community	43.4 (SD 7.7)	35.1%/ 64.9%	University employees	TG, HDL-c	TG ≥1.7, HDL-c <1.0 & 1.3	Low
Chan, WK[3]	2014	37	11, 8, 8, 9 - TC, LDL-c, TG, HDL-c	29.7%, 21.6%, 21.6%, 24.3% -TC, LDL-c, TG, HDL-c	Urban	Community	25.2 (SD 4.5)	75.7%/ 24.3%	Medical students, NAFLD	TC, LDL-c, TG, HDL-c	TC ≥5.2, LDL-c ≥3.4, TG ≥1.7, HDL-c<1 & <1.3	High
Cheah, WL[4]	2011	238	50 -TC	21% -TC	Rural	Community	49.9	46.5%/ 53.5%	No	TC	TC >5.2	Low
Daud, A[5]	2018	65	16 -TG	24.6% -TG	Suburban	Community	33.88 (SD 10.17)	32.3%/ 67.7%	Obese Malays	TG	TG ≥1.7	High
Goh, SC[6]	2012	368	98, 242 - TG, HDL-c	26.6%, 65.8% - TG, HDL-c	Suburban	Hospital-based	not stated	not stated	NAFLD, health screening	TG, HDL-c	TG ≥1.7, HDL-c <1.03 & 1.29	Low
Harris, H[7]	2019	330	140 -TC	42.4% -TC	Rural	Community	43.7 (SD 15.8)	40.3%/ 59.7%	Coastal communities	TC	TC ≥5.2	Low
Hejazi, N[8]	2013	2739	857, 555, 933, 454 - TC, LDL-c, TG, HDL-c	54.2%, 35.1%, 59%, 28.7% - TC, LDL-c, TG, HDL-c	Urban	Hospital-based ID clinic	not stated	81.1%/ 18.9%	HIV on ART	TC, LDL-c, TG, HDL-c	TC ≥5.17, LDL-c ≥3.36, TG ≥1.7, HDL-c<1.03 & <1.3	Low

Ismail, IS[9]	2001	848	588, 741, 177, 376 - TC, LDL-c, TG, HDL-c	69.3%, 87.4%, 20.9%, 44.3% -TC, LDL-c, TG, HDL-c	Urban	Hospital-based	not stated	47.9%/ 52.1%	Diabetes type 1 & type 2	TC, LDL-c, TG, HDL-c	TC ≥5.2, LDL-c ≥2.6, TG ≥2.3, HDL-c ≤ 1.15	Low
Khammas, ASA[10]	2019	628	411, 558, 175, 65 - TC, LDL-c, TG, HDL-c	65.4%, 88.9%, 27.9%, 10.4% -TC, LDL-c, TG, HDL-c	Urban	Clinic	54.54 (SD 6.69)	48.1%/ 51.9%	Health screening	TC, LDL-c, TG, HDL-c	TC ≥5.17, LDL-c ≥2.59, TG ≥1.7, HDL-c <1.04 & <1.3	Low
Khoo, KL[11]	1997	1116	654, 724, 165, 232 - TC, LDL-c, TG, HDL-c	58.6%, 64.9%, 14.8%, 20.8% -TC, LDL-c, TG, HDL-c	Urban	Clinic	43.6	74.2%/ 25.8%	Medical check up	TC, LDL-c, TG, HDL-c	TC ≥5.2, LDL-c ≥3.3, TG ≥2.3, HDL-c <0.9	Low
Liew, YM[12]	1997	719, 701, 718, 715 - TC, LDL-c, TG, HDL-c	541, 523, 143, 48 - TC, LDL-c, TG, HDL-c	75.2%, 74.6%, 19.9%, 6.7% -TC, LDL-c, TG, HDL-c	Urban	Community	44 (range 25-56)	70.9%/ 29.1%	Senior civil servants	TC, LDL-c, TG, HDL-c	TC ≥5.2, LDL-c ≥3.3, TG ≥2.3, HDL-c <0.9	Low
Lim, TO[13]	2000	17392	3496 -TC	20.1% -TC	Both	Community	not stated	47%/ 53%	No	TC	TC ≥5.2	Low
M Eid[14]	2004	211	148, 184, 96, 121 - TC, LDL-c, TG, HDL-c	70%, 87%, 46%, 57% - TC, LDL-c, TG, HDL-c	Urban	Hospital-based OPD clinic	53.65 (SD 9.53)	48%/ 52%	Diabetes	TC, LDL-c, TG, HDL-c	TC ≥5.2, LDL-c ≥2.6, TG ≥1.71, HDL-c ≤1.15 & ≤1.4	Low
Mohamed, M 2003[15]	2003	348	246 -TC	70.7% -TC	Both	Community	54.9 (SD 12.4)	29.0%/ 71.0%	Hypertension	TC	TC ≥5.2	Low
Mohamed, M 2005[16]	2005	438	384, 220, 302 -TC, TG, HDL-c	87.7%, 50.2%, 69.1% -TC, TG, HDL-c	Urban	Clinic	54.1 (SD 11.0)	52.0%/ 48.0%	Diabetes type 1 & type 2	TC, TG, HDL-c	TC ≥4.8, TG ≥1.7, HDL-c ≤1.2	High

Mohamed, M 2006[17]	2006	1099	747, 537, 444 -TC, TG, HDL-c	68.0%, 48.9%, 40.4% -TC, TG, HDL-c	Urban	Hospital-based diabetes clinics	55.8 (SD 11.4)	46.5%/ 53.5%	Diabetes type 1 & type 2	TC, TG, HDL-c	TC ≥4.8, TG ≥1.7, HDL-c ≤1.2	High
Mohamed, M 2011[18]	2011	1549	712, 307, 424 -LDL-c, TG, HDL-c	46.0%, 19.8%, 27.4% - LDL-c, TG, HDL-c	Urban	Hospital-based	57.5 (SD 10.9)	45.7%/ 51.3%	Diabetes type 2	LDL-c, TG, HDL-c	LDL-c >2.6, TG >2.2, HDL-c <1.0	Low
Mohamed, M 2016[19]	2016	1630, 1568, 1617, 1575 -TC, LDL-c, TG, HDL-c	422, 668, 327, 299 - TC, LDL-c, TG, HDL-c	25.9%, 42.6%, 20.2%, 19.0% -TC, LDL-c, TG, HDL-c	Urban	Hospital-based	57.8 (SD 11.0)	45.4%/ 54.6%	Diabetes type 2	TC, LDL-c, TG, HDL-c	TC >5.2, LDL-c >2.6, TG >2.2, HDL-c <1.0	Low
Mohamed-Yassin, MS[20]	2021	9704, 8976, 8978, 8981, 8981 -TC, LDL-c, TG, HDL-c, non-HDL-c	6226, 5010, 3355, 3245, 5061-TC, LDL-c, TG, HDL-c, non-HDL-c	64.0%, 56.7%, 37.4%, 36.2%, 56.2%	Both	Community	52.7 (SD 11.1)	43.3%/ 56.7%	No	TC, LDL-c, TG, HDL-c, non-HDL-c	TC >5.2, LDL-c >3.4, TG >1.7, HDL-c <1 & <1.2, non-HDL-c >4.2	Low
Mohd Zainuddin, LR[21]	2011	298	86, 152 - TG, HDL-c	28.9%, 51.0% -TG, HDL-c	Rural	Community	Range 18-70	41.7%/ 58.3%	No	TG, HDL-c	TG ≥1.7, HDL-c <1.03 & 1.29	High
Nawawi, H[22]	2002	609, 547, 597, 597 - TC, LDL-c, TG, HDL-c	410, 313, 275, 78 - TC, LDL-c, TG, HDL-c	67.3%, 57.2%, 46.1%, 0.9% -TC, LDL-c, TG, HDL-c	Rural	Community	44.5 (SD 9.1)	43.2%/ 56.8%	No	TC, LDL-c, TG, HDL-c	TC >5.2, LDL-c >3.4, TG >1.7, HDL-c <0.9	Low
Phipps, ME[23]	2015	636	152, 291 - TG, HDL-c	23.9%, 45.8% -TG, HDL-c	Rural	Community	Median 31 (range 18-80)	43.2%/ 56.8%	Indigenous	TG, HDL-c	TG ≥1.7, HD L<1 & 1.3	Low
Rabia, K[24]	2007	200	173, 176, 139, 39 - TC, LDL-c, TG, HDL-c	86.5%, 88.0%, 69.5%, 19.5% -TC, LDL-c, TG, HDL-c	Urban	Hospital-based primary care clinic	61.1 (SD 9.82)	39.5%/ 60.5%	Diabetes type 1 & type 2	TC, LDL-c, TG, HDL-c	TC >5.2, LDL-c >2.6, TG >1.7, HDL-c <1.0	High

Shafei, MN[25]	2007	148	56,51,51,17 -TC, LDL-c, TG, HDL-c	37.8%, 34.5%, 34.5%, 11.5% - TC, LDL-c, TG, HDL-c	Urban	Community	31.6 (SD 4.73)	not stated	Male factory workers	TC, LDL-c, TG, HDL-c	TC ≥6.22, LDL-c ≥4.14, TG ≥1.7, HDL-c ≤1.04	Low
Wan Mohamud, WN[26]	2012	4341	1611, 1853 -TG, HDL-c	37.1%, 42.7% -TG, HDL-c	Both	Community	47.8 (SD 14.5)	35.1%/ 64.9%	No	TG, HDL-c	TG ≥1.7, HDL-c <1.03 & 1.3	Low

eTable 4. Characteristics of Included Studies

Dyslipidaemia subtype	Prevalence (%) Pre sensitivity analysis			Prevalence (%) Post sensitivity analysis (Excluded study/studies with high risk of bias)		
	Community based studies	Hospital/ Clinic-based studies	Overall	Community based studies	Hospital/ Clinic-based studies	Overall
Elevated TC	48	63	53	50	56	52
Elevated LDL-c	-	73	73	-	69	69
Elevated TG	31	43	36	33	33	33
Low HDL-c	40	39	40	36	-	37

eTable 5. Sensitivity Analyses Findings Summary

Quality assessment						
**Study design**	**Risk of bias**	**Inconsistency**	**Indirectness**	**Imprecision**	**Publication bias**	**Quality of evidence**
Observational studies	Serious*	Very serious†	Serious‡	Serious§	Likely¶	⊕⊕ LOW

eTable 6. GRADE assessment of the studies included in meta-analyses

*Study quality assessed using the JBI Critical Appraisal Checklist for Studies Reporting Prevalence Data ranged from low to high risk of bias. Only 6 studies included in this meta-analysis were judged as having high risk of bias.
†Based on significant heterogeneity (I^2 ranged from 90 to 100%).
‡Indirectness - The study population can be generalized to the population of interest.
§Only very few studies had large 95% CIs.
¶Doi plots and LFK indices were consistent with the presence of publication bias.

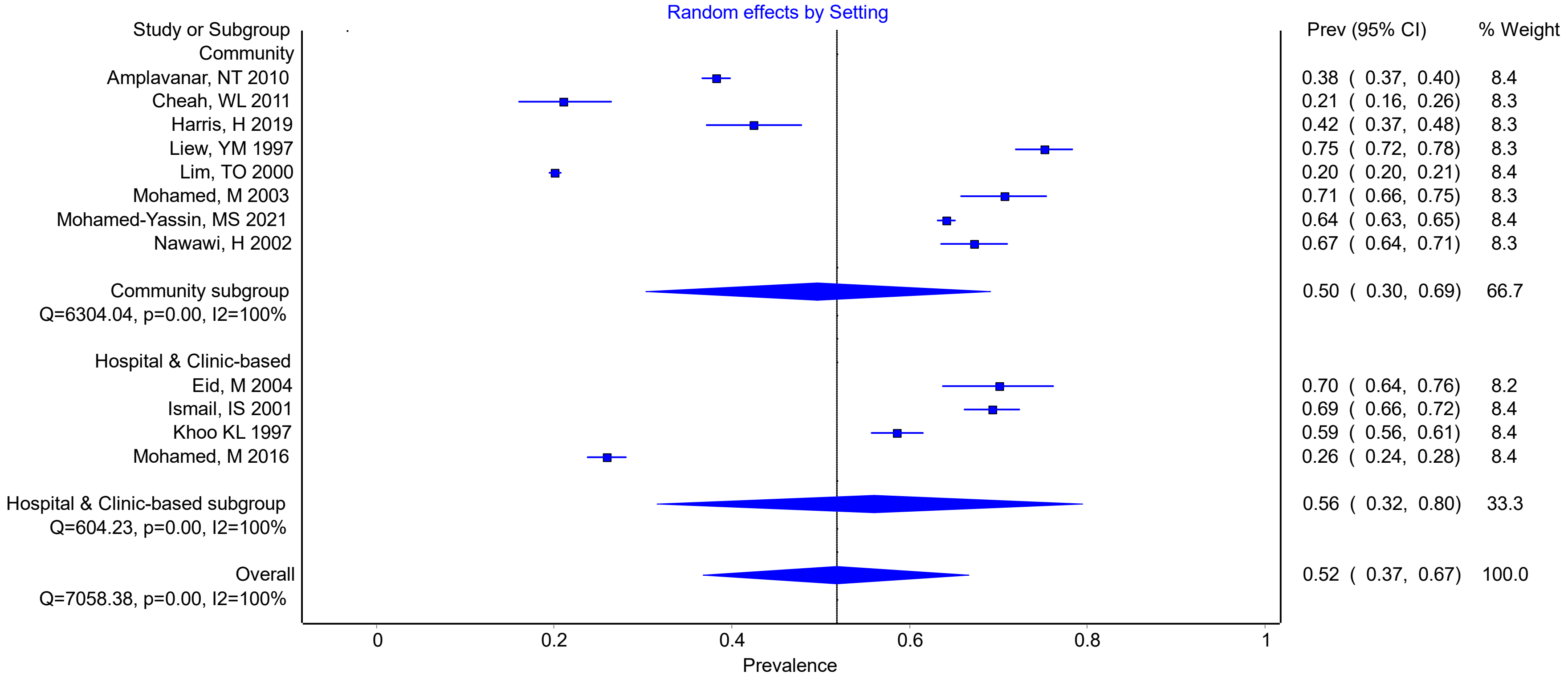

eFigure 1. Sensitivity analysis - Forest Plot Showing Prevalence of Elevated Total Cholesterol (TC ≥5.2 AND >5.2) in Community-based Studies and Hospital or Clinic-based Studies Excluding Studies with High Risk of Bias (Chan WK 2014 and Rabia K 2007)

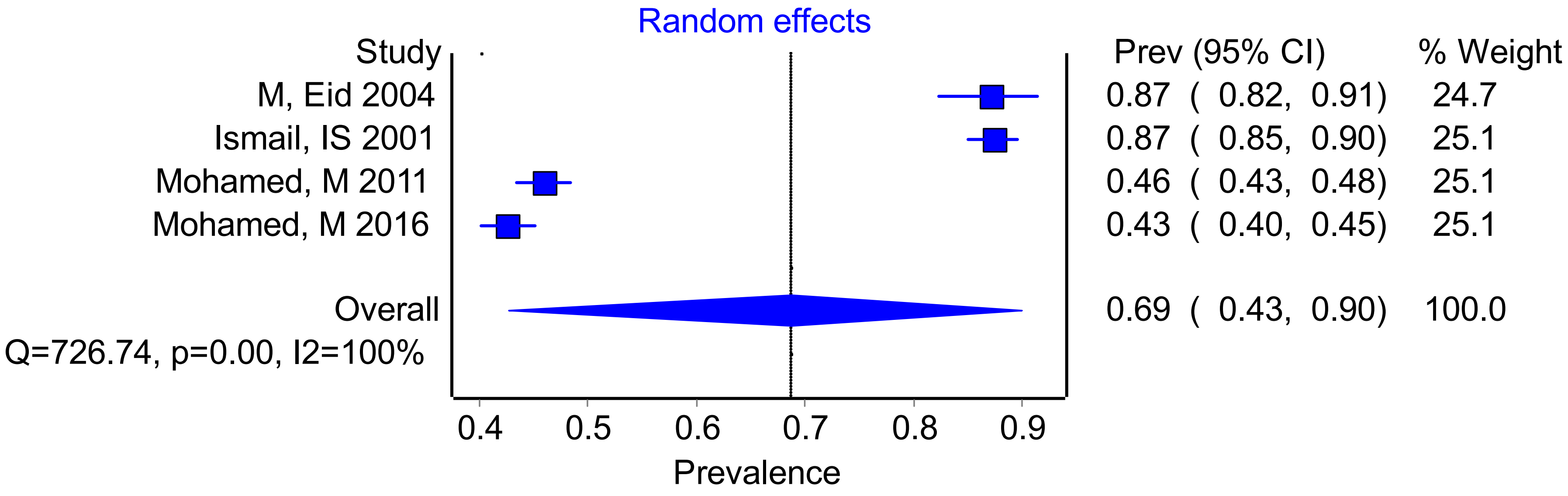

eFigure 2. Sensitivity analysis - Forest Plot Showing Prevalence of Elevated LDL-cholesterol (LDL-c ≥2.6) in Hospital or Clinic-based Studies Excluding Study with High Risk of Bias (Rabia K 2007)

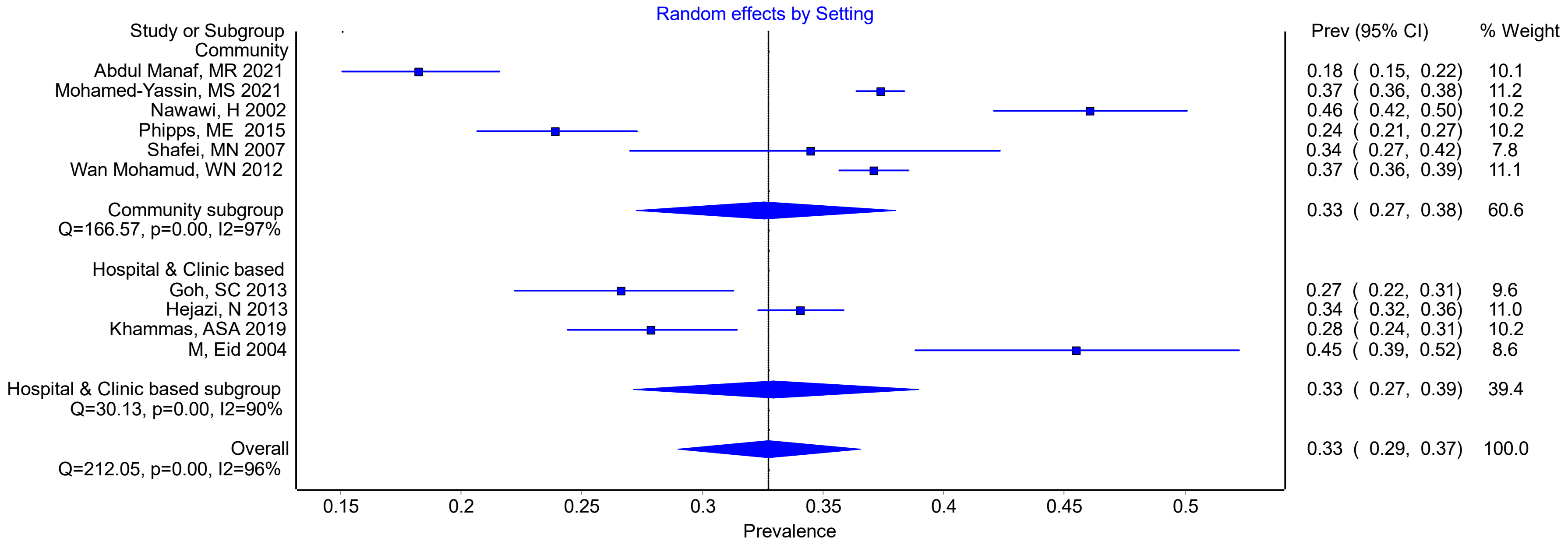

eFigure 3. Sensitivity analysis - Forest Plot Showing Prevalence of Elevated Triglycerides (TG ≥1.7 & >1.7) in Community-based Studies and Hospital or Clinic-based Studies Excluding Studies with High Risk of Bias (Rabia K 2007, Chan WK 2014, Daud A 2018, Mohamed M 2005, Mohamed M 2006, Mohd Zainuddin LR 2011)

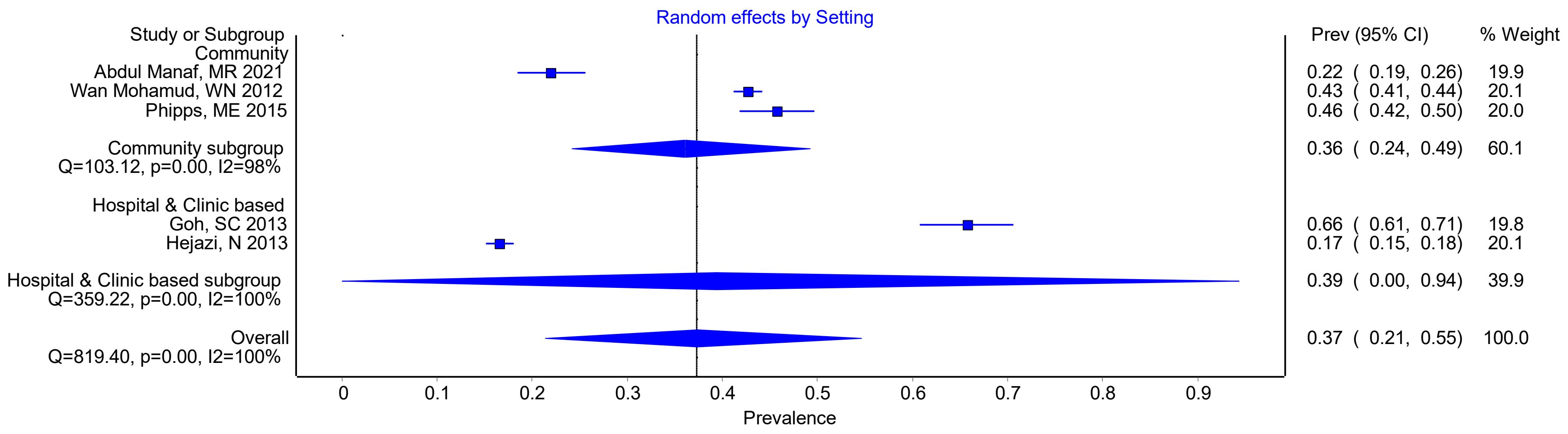

eFigure 4. Sensitivity analysis - Forest Plot Showing Prevalence of Low HDL-cholesterol (HDL-c <1 in men & <1.3 women) in Community-based Studies and Hospital or Clinic-based Studies Excluding Studies with High Risk of Bias (Mohd Zainuddin LR 2011)

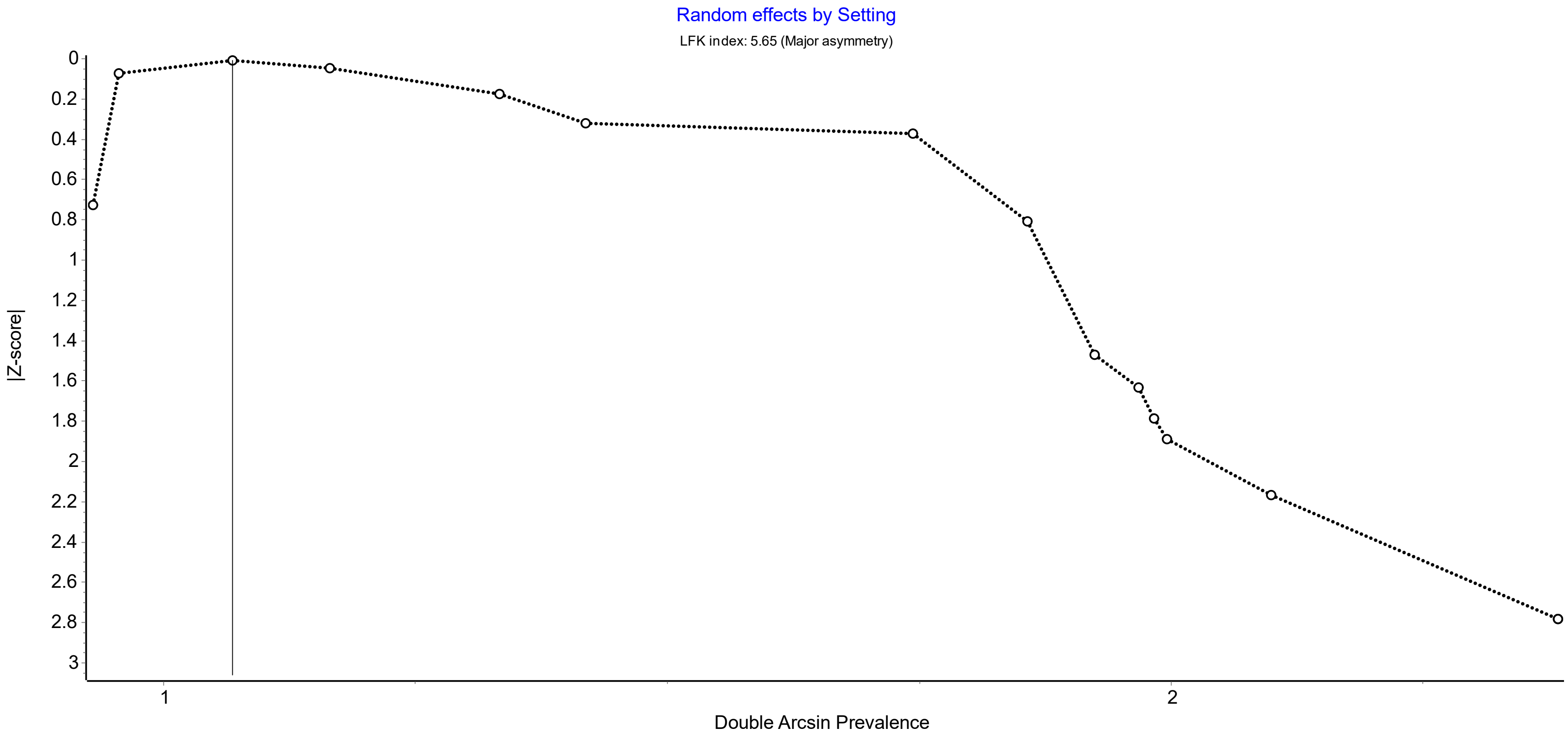

eFigure 5. Doi plot and LFK index for Elevated Total Cholesterol

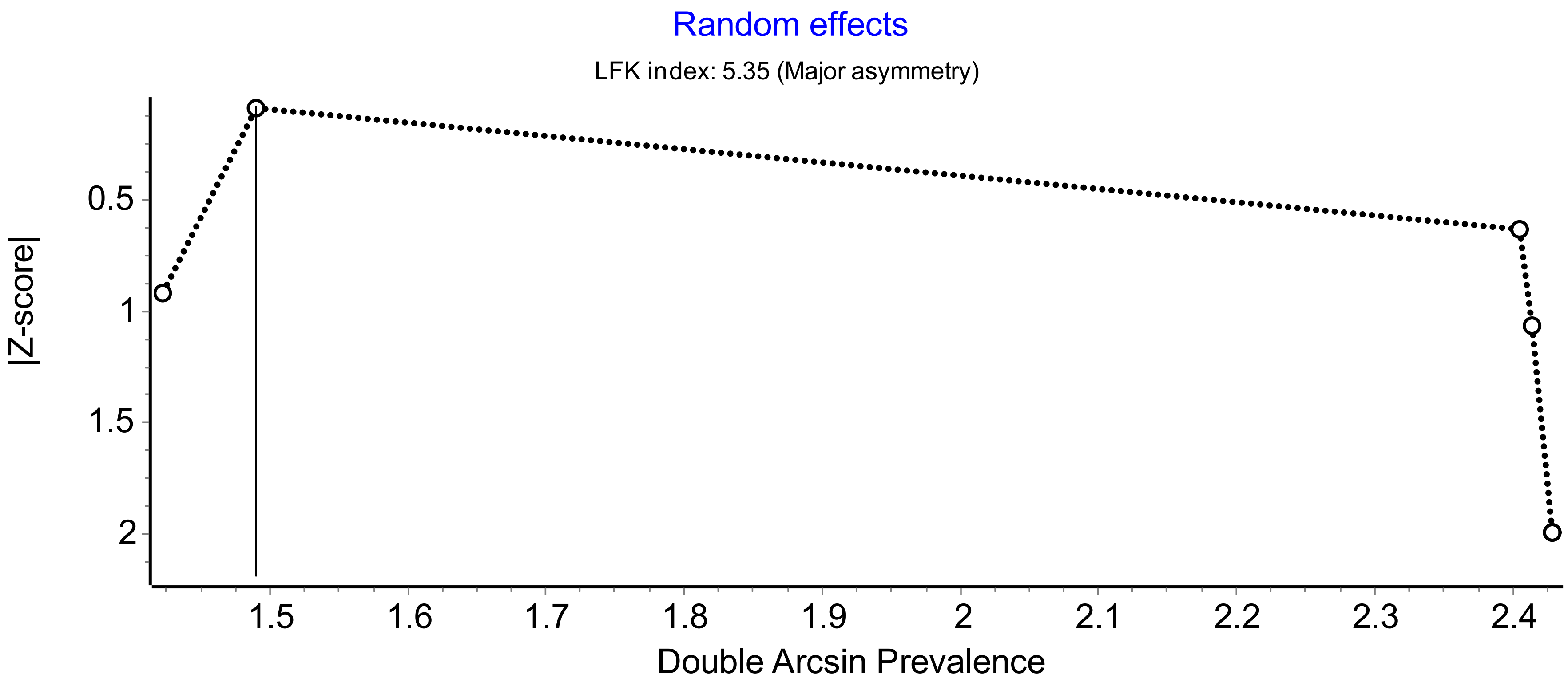

eFigure 6. Doi plot and LFK index for Elevated LDL-Cholesterol

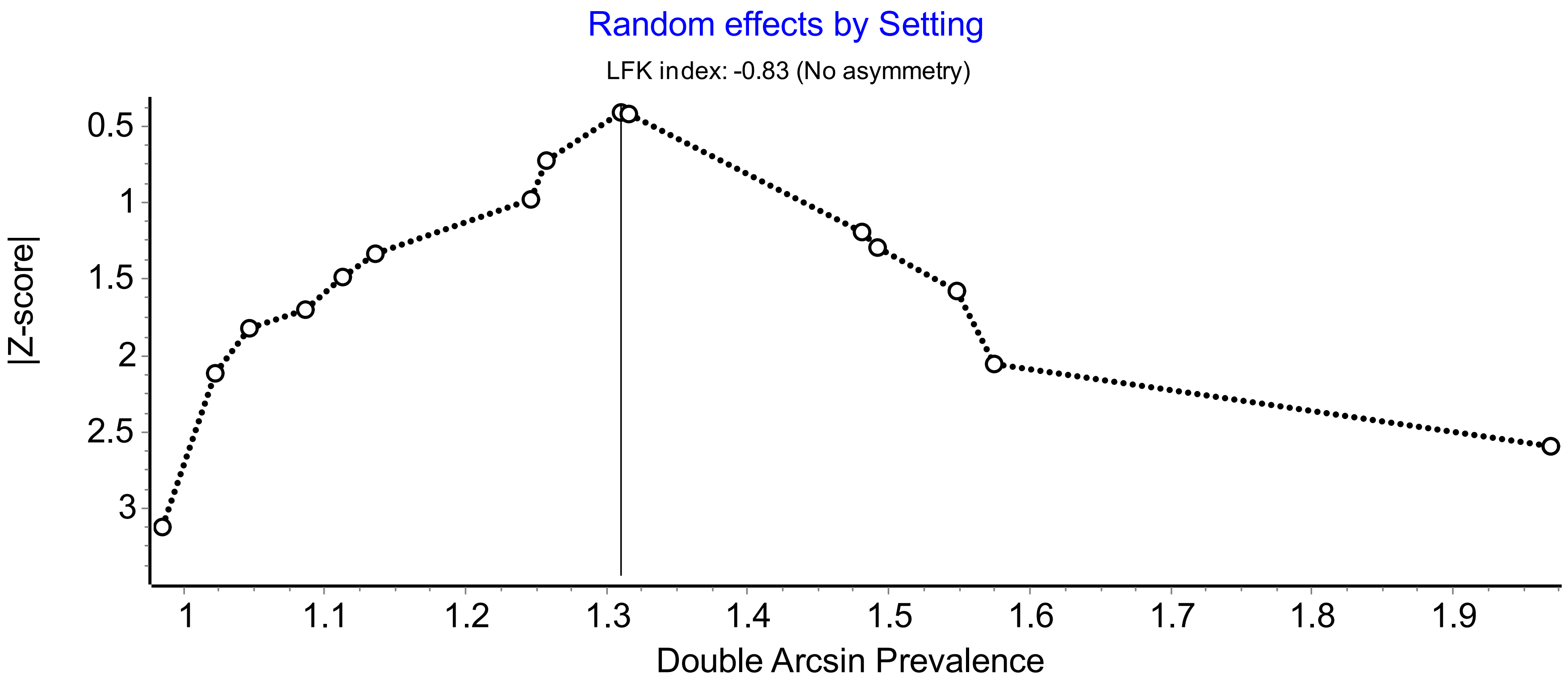

eFigure 7. Doi plot and LFK index for Elevated Triglycerides

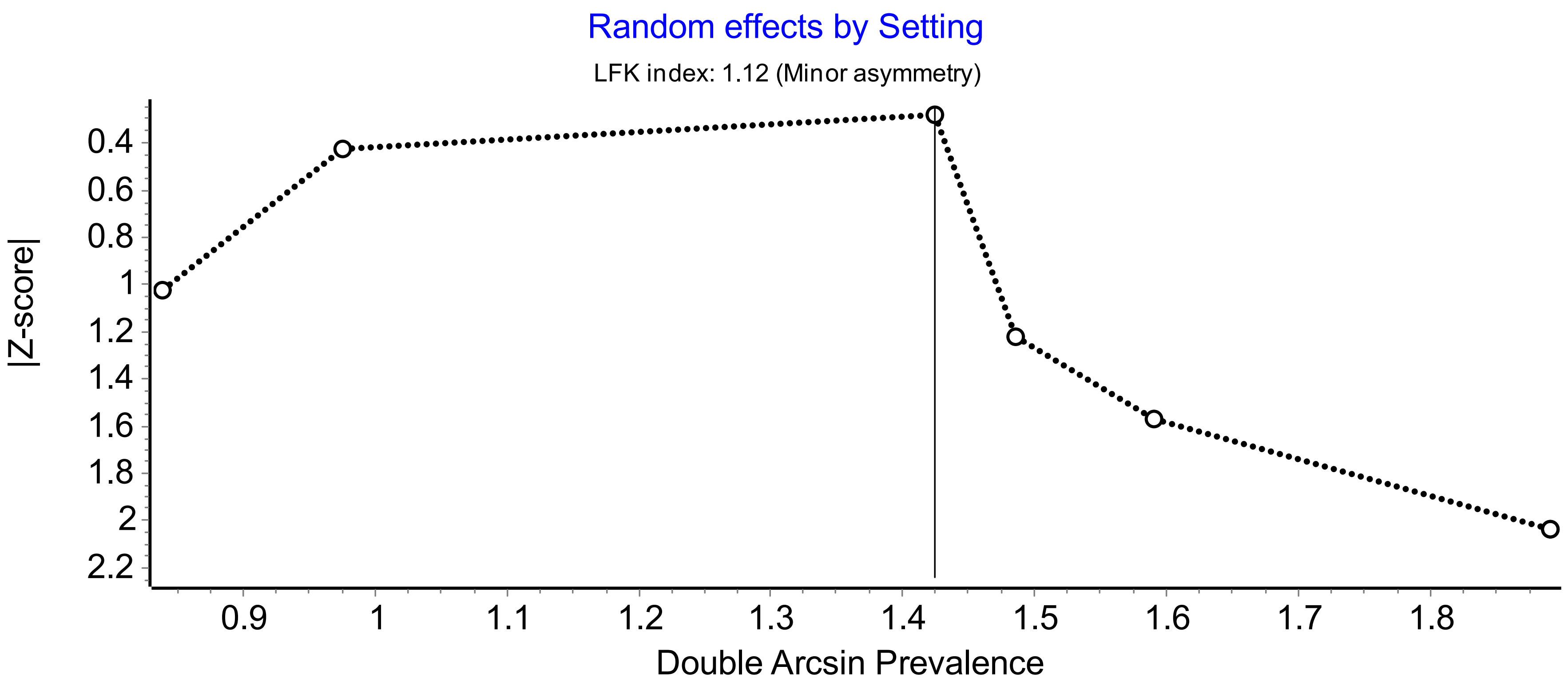

eFigure 8. Doi plot and LFK index for Low HDL-c

eReferences

1. Amplavanar NT, Gurpreet K, Salmiah MS, Odhayakumar N. Prevalence of cardiovascular disease risk factors among attendees of the Batu 9, Cheras Health Centre, Selangor, Malaysia. *Med J Malaysia.* 2010;65(3):173-179.
2. Manaf MRA, Nawi AM, Tauhid NM, et al. Prevalence of metabolic syndrome and its associated risk factors among staffs in a Malaysian public university. *Sci Rep.* 2021;11(1):8132.
3. Chan WK, Bahar N, Razlan H, Vijayananthan A, Sithaneshwar P, Goh KL. Non-alcoholic fatty liver disease in a young multiracial Asian population: a worrying ethnic predilection in Malay and Indian males. *Hepatol Int.* 2014;8(1):121-127.
4. Cheah WL, Lee PY, Khatijah Y, Rasidah AW. A preliminary study on the prevalence of cardiovascular disease risk factors in selected rural communities in Samarahan and Kuching division, Sarawak, Malaysia. *Malays J Med Sci.* 2011;18(2):58-65.
5. Daud A, Shahadan SZ, Ibrahim M, Lokman Md Isa M, Deraman S. Prevalence and association between triglyceride level and lifestyle factors among Malay obese class I and II adults. *Enferm Clin.* 2018;28 Suppl 1:310-315.
6. Goh SC, Ho EL, Goh KL. Prevalence and risk factors of non-alcoholic fatty liver disease in a multiracial suburban Asian population in Malaysia. *Hepatol Int.* 2013;7(2):548-554.
7. Harris H, Ooi YBH, Lee JS, Matanjun P. Non-communicable diseases among low income adults in rural coastal communities in Eastern Sabah, Malaysia. *BMC Public Health.* 2019;19(Suppl 4):554.
8. Hejazi N, Rajikan R, Choong CL, Sahar S. Metabolic abnormalities in adult HIV infected population on antiretroviral medication in Malaysia: a cross-sectional survey. *BMC Public Health.* 2013;13:758.
9. Ismail IS, Nazaimoon W, Mohamad W, et al. Ethnicity and glycaemic control are major determinants of diabetic dyslipidaemia in Malaysia. *Diabet Med.* 2001;18(6):501-508.
10. Khammas ASA, Hassan HA, Salih SQM, et al. Prevalence and risk factors of sonographically detected non alcoholic fatty liver disease in a screening

centre in Klang Valley, Malaysia: an observational cross-sectional study. *Porto Biomed J.* 2019;4(2):e31.

11. Khoo KL, Tan H, Liew YM. Serum lipids and their relationship with other coronary risk factors in healthy subjects in a city clinic. *Med J Malaysia.* 1997;52(1):38-52.
12. Liew YM, Zulkifli A, Tan H, Ho YN, Khoo KL. Health status of senior civil servants in Kuala Lumpur. *Med J Malaysia.* 1997;52(4):348-366.
13. Lim TO, Ding LM, Zaki M, et al. Clustering of hypertension, abnormal glucose tolerance, hypercholesterolaemia and obesity in Malaysian adult population. *Med J Malaysia.* 2000;55(2):196-208.
14. Eid M, Mafauzy M, Faridah AR. Non-achievement of clinical targets in patients with type 2 diabetes mellitus. *Med J Malaysia.* 2004;59(2):177-184.
15. Mafauzy M, Mokhtar N, Wan Mohamad WB. Hypertension and associated cardiovascular risk factors in Kelantan. *Med J Malaysia.* 2003;58(4):556-564.
16. Mafauzy M. Diabetes control and complications in private primary healthcare in Malaysia. *Med J Malaysia.* 2005;60(2):212-217.
17. Mafauzy M. Diabetes control and complications in public hospitals in Malaysia. *Med J Malaysia.* 2006;61(4):477-483.
18. Mafauzy M, Hussein Z, Chan SP. The status of diabetes control in Malaysia: results of DiabCare 2008. *Med J Malaysia.* 2011;66(3):175-181.
19. Mafauzy M, Zanariah H, Nazeri A, Chan SP. DiabCare 2013: A cross-sectional study of hospital based diabetes care delivery and prevention of diabetes related complications in Malaysia. *Med J Malaysia.* 2016;71(4):177-185.
20. Mohamed-Yassin MS, Baharudin N, Daher AM, et al. High prevalence of dyslipidaemia subtypes and their associated personal and clinical attributes in Malaysian adults: the REDISCOVER study. *BMC Cardiovasc Disord.* 2021;21(1):149.
21. Zainuddin LR, Isa N, Muda WM, Mohamed HJ. The prevalence of metabolic syndrome according to various definitions and hypertriglyceridemic-waist in malaysian adults. *Int J Prev Med.* 2011;2(4):229-237.
22. Nawawi HM, Nor IM, Noor IM, et al. Current status of coronary risk factors among rural Malays in Malaysia. *J Cardiovasc Risk.* 2002;9(1):17-23.

23. Phipps ME, Chan KK, Naidu R, et al. Cardio-metabolic health risks in indigenous populations of Southeast Asia and the influence of urbanization. *BMC Public Health.* 2015;15:47.
24. Rabia K, Khoo EM. Prevalence of peripheral arterial disease in patients with diabetes mellitus in a primary care setting. *Med J Malaysia.* 2007;62(2):130-133.
25. Nazri SM, Tengku MA, Winn T. Lipid disorders among male factory shift workers in Kota Bharu, Kelantan. *Med J Malaysia.* 2007;62(2):134-138.
26. Mohamud WN, Ismail A, Khir AS, et al. Prevalence of metabolic syndrome and its risk factors in adult Malaysians: results of a nationwide survey. *Diabetes Res Clin Pract.* 2012;96(1):91-97.
